# Nrf2-ARE-Dependent Alterations in Zinc Transporter mRNA Expression in HepG2 Cells

**DOI:** 10.1371/journal.pone.0166100

**Published:** 2016-11-03

**Authors:** Takumi Ishida, Shinji Takechi

**Affiliations:** Faculty of Pharmaceutical Sciences, Sojo University, 4-22-1 Ikeda, Nishi-ku, Kumamoto 860-0082, Japan; Hokkaido Daigaku, JAPAN

## Abstract

Zinc transporters are solute carrier family members. To date, 10 zinc transporters (ZnTs) and 14 Zrt-, Irt-like proteins (ZIPs) have been identified. ZnTs control intracellular zinc levels by effluxing zinc from the cytoplasm into the extracellular fluid, intracellular vesicles, and organelles; ZIPs also contribute to control intracellular zinc levels with influxing zinc into the cytoplasm. Recently, changes in zinc transporter expression have been observed in some stress-induced diseases, such as Alzheimer’s disease and diabetes mellitus. However, little is known regarding the mechanisms that regulate zinc transporter expression. To address this, we have investigated the effect of a well-established stress response pathway, the nuclear factor erythroid 2-related factor 2 (Nrf2)-antioxidant responsive element (ARE) pathway, on zinc transporter mRNA levels. Exposure to 10^−4^ M *tert*-butylhydroquinone (*t*-BHQ), which activates Nrf2-ARE signaling, for 6 h significantly increases ZnT-1, ZnT-3, and ZnT-6 mRNAs levels, and significantly decreases ZnT-10 and ZIP-3 mRNA levels. These changes are not observed with 10^−6^ M *t*-BHQ, which does not activate Nrf2-ARE signaling. Furthermore, *t*-BHQ exposure does not affect metal responsive element transcription, a *cis* element that is activated in response to intracellular free zinc accumulation. From these results, we believe that the transcription of ZnT-1, ZnT-3, ZnT-6, ZnT-10, and ZIP-3 is influenced by the Nrf2-ARE signal transduction pathway.

## Introduction

Zinc is an essential trace element in humans, and it is estimated that there is approximately 2 g of zinc in the human body. The majority of *in vivo* zinc is located in the skeletal muscle and bone (approximately 60% and 30%, respectively), and the rest is distributed in widespread tissues such as the liver, prostate gland, and skin [1]. Zinc is also present in the brain; it is especially concentrated in the hippocampus and amygdala [2], where it is found as a free ionic form (Zn^2+^) within synaptic vesicles [3]. Zinc plays a unique roles in many biological processes, including cell division, growth, and differentiation, and serves as a structural, catalytic, and regulatory component of proteins, such as transcription factors, enzymes, transporters, and receptors [4–6]. Intracellular free zinc levels need to be efficiently controlled by the homeostatic system to avoid toxicity associated with excess zinc accumulation or deficiency. Zinc homeostasis is regulated through zinc transporters, permeable channels and metallothioneins, although zinc transporters are considered to be most significant in controlling intracellular free zinc levels. Zinc transporters are secondary active transporters, which do not consume adenosine triphosphate, and efflux and influx zinc from the cytoplasm to the extracellular fluid, intracellular vesicles, and organelles. To date, 10 zinc transporters (ZnTs) and 14 Zrt-, Irt-like proteins (ZIPs) have been reported to function as zinc transporters. ZnTs (also known as SLC30A subfamily members) function to reduce intracellular zinc availability by promoting zinc efflux from cytoplasm or into intracellular vesicles, while ZIPs (also known as SLC39A subfamily members) function to increase intracellular zinc by promoting extracellular zinc uptake and the release of vesicular zinc into the cytoplasm [7,8]. Recently, the relationship between the expression/activity of zinc transporters and disease has attracted considerable attention. For example, ZnT-10 mRNA expression is decreased in the frontal cortex of Alzheimer’s disease patients [9], ZIP-4 is dysfunctional in acrodermatitis enteropathica [10,11], and ZnT-8 mutant allele are associated with an increased risk of type 2 diabetes mellitus [12]. These studies suggest that altered zinc transporter expression and activity can contribute to disease onset. As such, a better understanding of the transcriptional mechanisms regulating zinc transporter expression could help our understanding of disease pathogenesis.

The nuclear factor erythroid 2-related factor 2 (Nrf2)-antioxidant responsive element (ARE) signal transduction pathway is a well-documented stress response pathway in mammalian cells. The Nrf2-ARE pathway is activated by oxidative stress and electrophilic chemicals, and regulates the transcription of numerous stress response proteins. It is widely accepted that excess stress is a fundamental cause of various diseases, including cancer, arteriosclerosis, diabetes mellitus, and Alzheimer’s disease. Because alterations in zinc transporter expression have also been suggested to participate in the onset of disease, it is possible that zinc transporter transcription is regulated by the Nrf2-ARE signal transduction pathway. Nevertheless, our current understanding of the mechanisms underlying zinc transporter transcription is quite poor. To address this, we have investigated Nrf2-ARE signaling-dependent alterations in zinc transporter mRNA levels in HepG2 cells, using *tert*-butylhydroquinone (*t*-BHQ) as an activator of the Nrf2-ARE signaling pathway. Although *t*-BHQ is known as a pro-oxidant [13,14], it can induce phase II detoxification enzymes, such as glutathione-*S*-transferase, via the Nrf2-ARE signal transduction pathway [13,15–17]. As such, *t*-BHQ can serve as a useful compound to improve our understanding of the cellular effects through the Nrf2-ARE signal transduction pathway.

## Materials and Methods

### Cell treatments and reagents

The HepG2 human hepatocellular carcinoma cell line was obtained from the Human Science Research Resources Bank (JCRB1054; Osaka, Japan). Cells were cultured in Dulbecco’s modified Eagle’s medium supplemented with 10% (v/v) fetal bovine serum in a humidified atmosphere at 37°C with 5% CO_2_. Prior to treatment, cells were grown in 60-mm culture dishes to 80–90% confluence. *t*-BHQ was purchased from WAKO Pure Chemical Industries, Ltd. (Osaka, Japan). The *t*-BHQ stock solution was prepared with 10% dimethyl sulfoxide, and HepG2 cells were treated with culture medium containing 1% (v/v) *t*-BHQ stock solution. Unless stated otherwise, all of the reagents and chemicals used were of the highest commercially available grade.

### RNA extraction and quantitative real-time polymerase chain reaction (qRT-PCR)

Total RNA was extracted from HepG2 cells using the RNeasy^®^ Mini Kit (Qiagen, GmbH, Hilden, Germany) according to the manufacturer’s instructions. The RNA concentration and purity were determined spectrophotometrically. Contaminating DNA was digested and reverse transcription was performed using approximately 0.1 μg of total RNA and the ReverTraAce^®^ qPCR RT Master Mix with gDNA Remover (Toyobo Co., Ltd., Osaka, Japan). The contamination of DNA in template was checked by the PCR using ß-actin primers bracketing an intron sequence, and we ascertained that the product derived from the primers was single and appropriate size (Table 1). The qRT-PCR assays were performed on a MiniOpticon^™^ real-time PCR system (Bio-Rad Laboratories, Inc., Hercules, CA, USA) using SYBR^®^ Green fluorescence detection and melting curve analysis. The primer sequences and estimated product sizes are shown in Table 1. Primers were designed using Prime3Plus web software [18], and the specificity of the primer sets was checked using BLAST^®^ (National Center for Biotechnology information, U.S. National Library of Medicine). Each 20 μL qRT-PCR reaction consisted of 10 μL of THUNDERBIRD^™^ SYBR^®^ qPCR Mix (Toyobo Co., Ltd.), 2 μL of 2.5 pmol/μL forward and reverse primers, and 2 μL of cDNA template. The PCR thermal cycling conditions were 95°C for 60 s, followed by 45 cycles of 95°C for 15 s and 60°C for 60 s. A final melting curve analysis (50–95°C, plate read/0.5°C, 5 s hold) was performed as an indicator of amplicon specificity. The Ct value, which corresponds to the number of reaction cycles required to detect a signal above baseline intensity, was calculated using Opticon Monitor^™^ software. The relative expression of each target mRNA was determined with reference to ß-actin expression.

**Table 1:** Quantitative real-time polymerase chain reaction (qRT-PCR) primer sequences and product sizes.

zinc transporters			
Symbol	Sequence (5’-3’)		Size (bp)
	Forward	Reverse	
ZnT-1	TGTGAACTTGCCTGCAGAAC	TGCTAACTGCTGGGGTCTTT	103
ZnT-2	CGGTCATACACGGGATCTCT	CTGTCAGGACCCTTCTGAGC	134
ZnT-3	GGAGTCCAAACCTGTGGAGA	CAGCCATGAAGACAAAGCAA	137
ZnT-4	TCTGGGTGTGAACGTAACCA	GATGGGGTCAGCAATCTTGT	150
ZnT-5	GGAGGACCAGCAAAGACAAG	CTTCAGGGTGTTCAGCCATT	109
ZnT-6	GAATTCGACGAGATGCCAAT	TAGGACATTGGCTGCAACAG	149
ZnT-7	CCCTGTCCATCAAAGACGAT	CCACAAAAGCGAAAGAGAGG	147
ZnT-8	GGCCAGCACCATCACTATCT	CCCCGTCGACTGCTAAAATA	110
ZnT-9	GTCATGGGATTGCTTCATCC	ATTCCTTTAGCCCGAGCATT	140
ZnT-10	GGTCCCAAAAGGAGTCAACA	GTGCAGGGTGGCAATAATCT	124
ZIP-1	GCCAGGAGCTAACCATGAAG	CCGCGAAACAGCTTACTAGG	68
ZIP-2	TCCACAGCCATGGACATTTA	CCACAGCTAGCCCTTCAAAC	105
ZIP-3	CCGTGAAGATCATCGAGACA	CTGGAGCTTTTCCCTCACAG	140
ZIP-4	TGGACGTTGTTGGACTTGAA	TGTGGGCAGAGACAAGTGAG	112
ZIP-5	GGGTGACCTGGAAGAGTCAA	GCCGTTCAGACAATCCTCAT	133
ZIP-6	CCCTCCCACTTTGATTCTCA	GCCGAGTGTATCGTGGAAAT	147
ZIP-7	ATACCCACGATCACGACCAT	CATGTCCATGTCCTCTGTGG	102
ZIP-8	TCCTGCACCTTGTCTCTCCT	AAGGCTTGTCGAGTGCTCAT	125
ZIP-9	GGGAGCAGCAGCATCTACTT	CAGCATGCATCAAGAAGGAA	113
ZIP-10	TCCTTGCTAGGCGTGATCTT	TGATCATGTCCACCCTGAGA	146
ZIP-11	AATCGGGATCCAGAATTTCC	CTGCCCATACCAGAAAGCTC	94
ZIP-12	ACAGAAGGGCCTCTCACTCA	GCTTGTTGGTCCTTGGGTAA	108
ZIP-13	CTGGCGGCTTTCTCTACATC	AGAGCGAGAACAGCACCATT	132
ZIP-14	AAGGCCCTACTCAACCACCT	CACGTGCTGGGTGACATTAC	60
Others			
ß-actin	CCTGGCACCCAGCACAAT	GGGCCGGACTCGTCATACT	144
MTF-1	CGAAGGAGAAGCCATTTGAG	ATTTGCTGCAGCCTTCAGAT	132
HMOX-1	TTCTCCGATGGGTCCTTACACT	GGCATAAAGCCCTACAGCAACT	62

The accession numbers used for primer design are shown in S1 Table.

### Reporter gene assays

pGL4 firefly luciferase reporter vectors encoding the ARE (pGL4.37[*luc2P/ARE/Hygro*]) and metal responsive element (MRE; pGL4.40[*luc2P/MRE/Hygro*]), and the pGL4 *Renilla* luciferase reporter vector (pGL4.74[*hRluc/TK*]) were obtained from Promega Corporation (Madison, WI, USA). To analyze ARE and MRE transcription, HepG2 cells were grown in 96-well plates for 24 h prior to transfection with 200 ng/well of either pGL4.37[*luc2P/ARE/Hygro*] or pGL4.40[*luc2P/MRE/Hygro*], and 20 ng/well of pGL4.74[*hRluc/TK*] using Hilymax (Dojin Molecular Technologies Inc., Kumamoto, Japan). After 24 h, the transfected cells were treated with the denoted concentrations of *t*-BHQ for 6 h. Cells were then lysed in Passive Lysis Buffer (Promega), and firefly and *Renilla* luciferase activities were determined using the Dual Luciferase Reporter Assay System (Promega) according to the manufacturer’s protocol. Luminescence was measured using an Infinite M200 PRO luminometer (Tecan Group Ltd., Männedorf, Switzerland), and firefly luciferase expression was corrected for transfection efficiency using *Renilla* luciferase activity.

### Cell viability assay

Cell viability was determined using a Cell Count Kit-8 (Dojin Molecular Technologies Inc.) according to the manufacturer’s instructions. Briefly, HepG2 cells were seeded in 96-well plates at a density of 1 × 10^4^ cells/well/100 μL medium and cultured overnight. After incubation with *t*-BHQ-containing medium, cells were washed with 150 μL phosphate-buffered saline, and new culture medium containing 10% (v/v) WST-8 solution was added to each well. Cells were incubated for 60 min in a CO_2_ incubator to obtain adequate WST-8 coloring, and absorbance was measured at 450 nm using a microtiter plate reader.

### Statistical analysis

Significant differences between two groups were calculated using the Student’s unpaired *t*-test. Significant differences among multiple groups were tested using the one-way analysis of variance, followed by the Dunnet’s post hoc multiple comparison test.

## Results

### Alteration of zinc transporter mRNA levels by *t*-BHQ

As a preliminary study, to evaluate the specificity of the designed primers, we performed PCR using cDNA derived from untreated HepG2 cells. Apart from ZnT-2, ZIP-2, ZnT-4, ZnT-8, and ZIP-12, single amplicon PCR products were detected for all of the zinc transporters. The size of the PCR products was in good agreement with the estimated value (Table 1). From these results and our BLAST analysis, we concluded that the designed primers specifically amplified the cDNA of each target zinc transporter.

**Fig 1:**
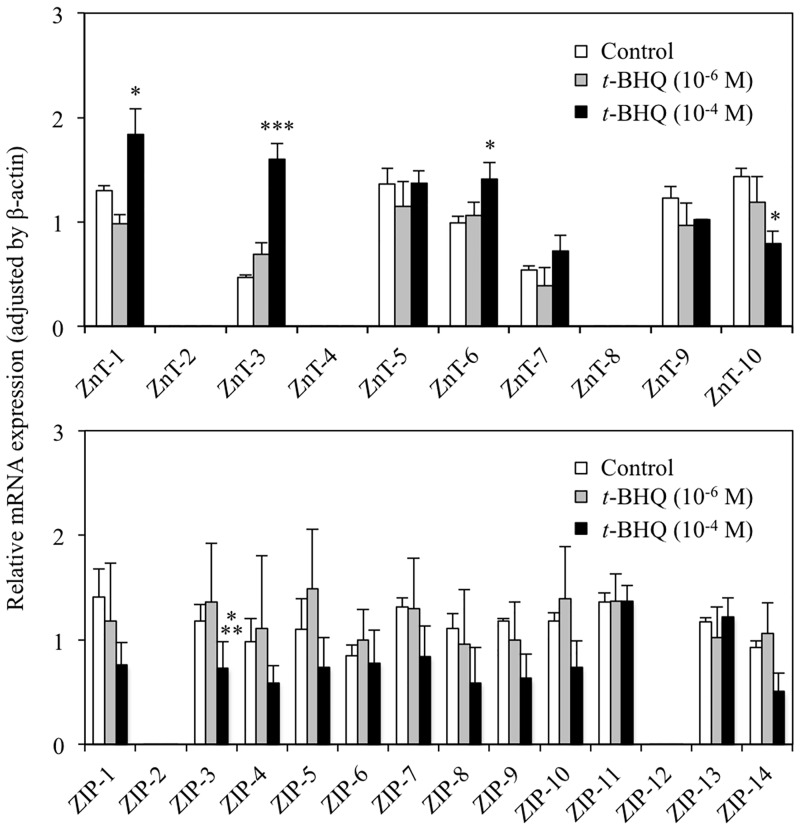
The effect of *tert*-butylhydroquinone (*t*-BHQ) treatment on zinc transporter mRNA levels. HepG2 cells were treated with the denoted concentration of *t*-BHQ for 6 h. The expression of each mRNA was determined by qRT-PCR with SYBR^®^ Green. Data are normalized to ß-actin mRNA expression. Values represent the mean ± standard deviation (SD) of 4 samples. *p < 0.05, ***p < 0.001 compared with vehicle treated control (0.1% dimethyl sulfoxide; DMSO) cells.

To determine the effect of Nrf2-ARE signaling on zinc transporter expression, qRT-PCR was performed using extracts from *t*-BHQ-treated HepG2 cells. *t*-BHQ concentrations of 10^−4^ M and 10^−6^ M were used as responsive and non-responsive doses, in accordance with the ARE-encoding luciferase reporter datasheet. As shown in Fig 1, apart from ZnT-2, ZIP-2, ZnT-4, ZnT-8, and ZIP-12, mRNA for all of the zinc transporters was detected in vehicle (dimethyl sulfoxide-treated) and *t*-BHQ-treated HepG2 cells. A significant increase in ZnT-1, ZnT-3, and ZnT-6 mRNA expression was observed after 6 h treatment with 10^−4^ M *t*-BHQ. Among these, ZnT-3 showed the greatest increase (approximately 3.4-fold) in response to *t*-BHQ; ZnT-1 and ZnT-6 both showed an approximately 1.4-fold increase in mRNA expression with *t*-BHQ treatment. In contrast, the expression of ZnT-10 and ZIP-3 mRNAs was significantly decreased, by approximately 50% and 40% respectively, with *t*-BHQ treatment. These significant alterations in zinc transporter mRNA expression were not observed with 10^−6^ M *t*-BHQ.

### Validation of *t*-BHQ-stimulated activation of Nrf2-ARE signaling

**Fig 2:**
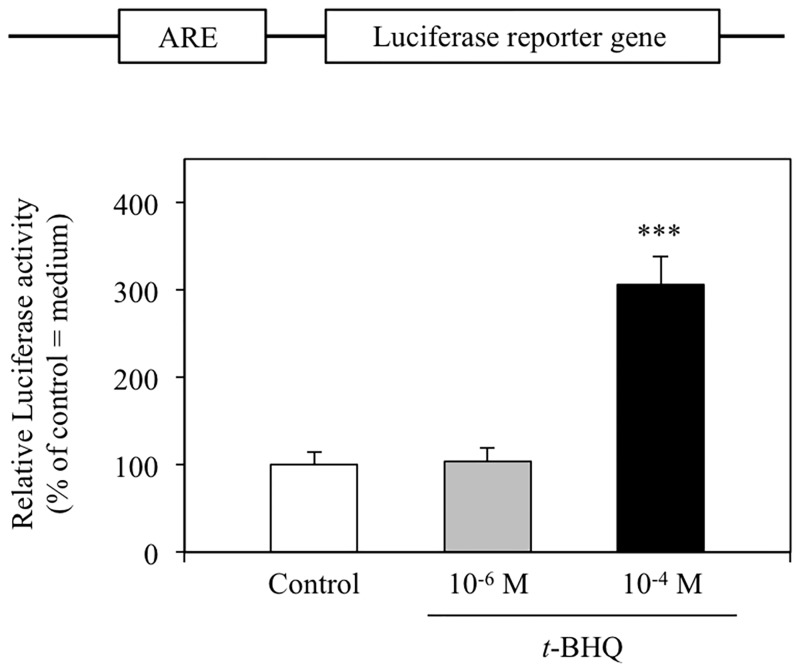
Antioxidant responsive element (ARE) luciferase reporter activity following *t*-BHQ treatment. HepG2 cells were cotransfected with pGL4.37, a firefly luciferase vector encoding the ARE, and the *Renilla* luciferase vector pGL4.74 prior to treatment with the denoted concentration of *t*-BHQ for 6 h. Firefly and *Renilla* luciferase activities were subsequently determined and firefly luciferase activity was normalized to the *Renilla* luciferase activity to control for transfection efficiency. Data are expressed as relative activity values compared with vehicle control (0.1% DMSO = 100%). Bars indicate the mean ± SD of 3 (10^−6^ M *t*-BHQ) or 4 (Control and 10^−4^ M *t*-BHQ) samples. ***p < 0.001 compared with control.

**Fig 3:**
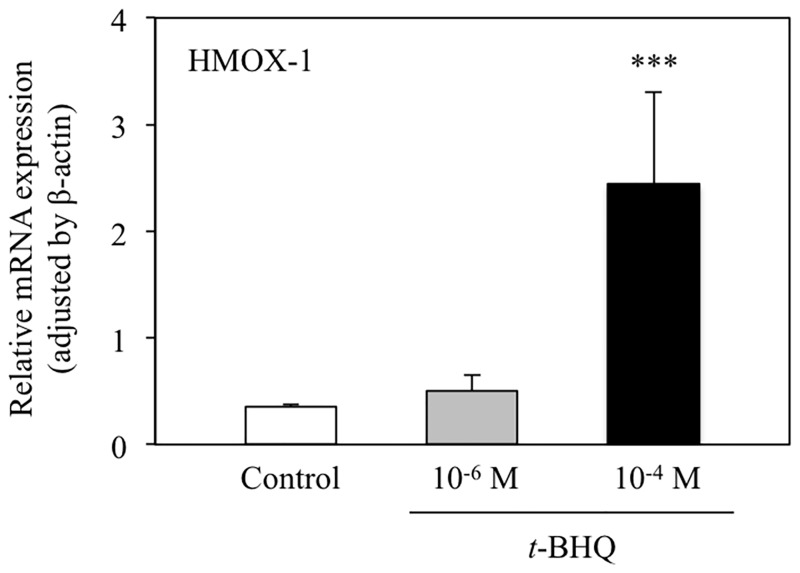
Dose-dependent alteration in heme oxygenase-1 mRNA expression following *t*-BHQ treatment. HepG2 cells were treated with the denoted concentration of *t*-BHQ or vehicle (0.1% (v/v) DMSO) for 6 h prior to quantification of mRNA expression by qRT-PCR with SYBR^®^ Green. Data are normalized to ß-actin expression. Bars indicate the mean ± SD of 4 samples. ***p < 0.001 compared with control.

**Fig 4:**
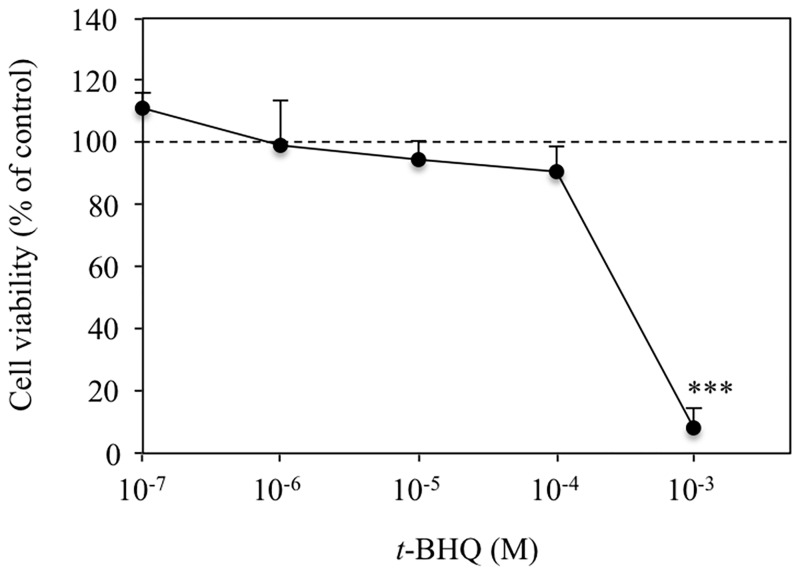
*t*-BHQ dose-dependent changes in HepG2 cell cytotoxicity. Cells were treated with the denoted concentration of *t*-BHQ or vehicle (0.1% (v/v) DMSO) for 6 h and cell viability was assessed using the WST-8 assay. Values represent the mean ± SD of 5 samples. ***p < 0.001 compared with control (100 ± 4.8%).

To confirm that *t*-BHQ activated Nrf2-ARE signaling in our experimental conditions, we performed a luciferase reporter assay using the ARE-encoding vector. In cells exposed to 10^−4^ M *t*-BHQ for 6 h, a significant, approximately 3-fold, increase in luciferase activity was detected (Fig 2). This increase in activity was not detected in cells treated with 10^−6^ M *t*-BHQ. Similarly, 10^−4^ M *t*-BHQ, but not 10^−6^ M *t*-BHQ, produced a significant increase in heme oxygenase-1 mRNA levels in HepG2 cells (Fig 3; HMOX-1). Heme oxygenase-1 is stress response gene induced by Nrf2-ARE signaling [19]. We believe that the adopted treatment condition of 10^−4^ M *t*-BHQ for 6 h efficiently activates Nrf2-ARE signaling without causing critical cell damage because cell viability was not significantly affected by incubation with 10^−4^ M to 10^−7^ M *t*-BHQ for 6 h (Fig 4).

### The effect of *t*-BHQ is independent of metal transcriptional factor-1 (MTF-1)-MRE signaling

**Fig 5:**
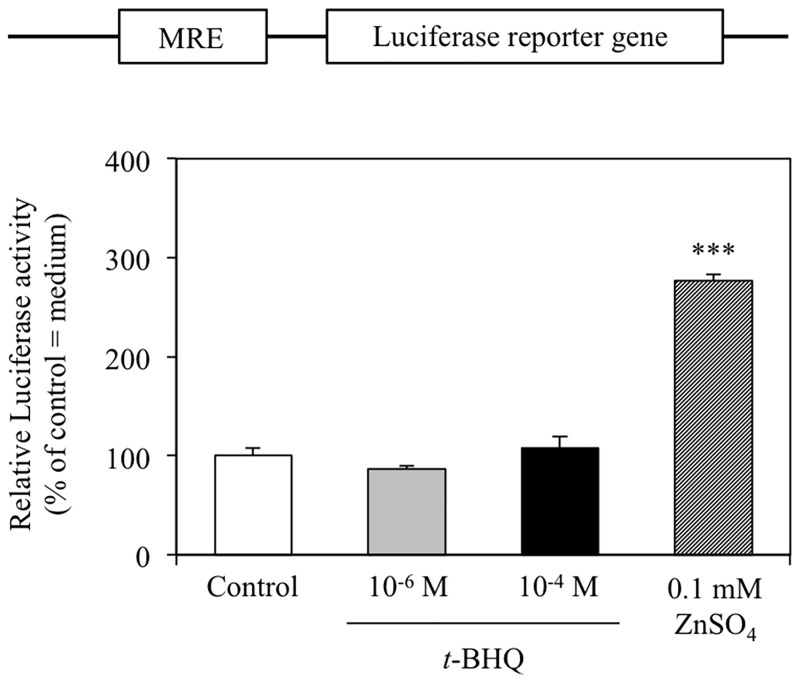
Metal responsive element (MRE)-luciferase reporter activity following *t*-BHQ treatment. HepG2 cells were cotransfected with the firefly luciferase vector encoding the MRE, pGL4.40, and the pGL4.74 *Renilla* luciferase vector. Transfected cells were treated with control (0.1% (v/v) DMSO), *t*-BHQ, or 0.1 mM ZnSO_4_ (as a positive control) for 6 h and luciferase activities were determined. Firefly luciferase activity was normalized to *Renilla* luciferase activity to control for transfection efficiency. Data are expressed as relative activity values compared with control (0.1% DMSO = 100%). Bars indicate the mean ± SD of 4 samples. ***p < 0.001 compared with control.

**Fig 6:**
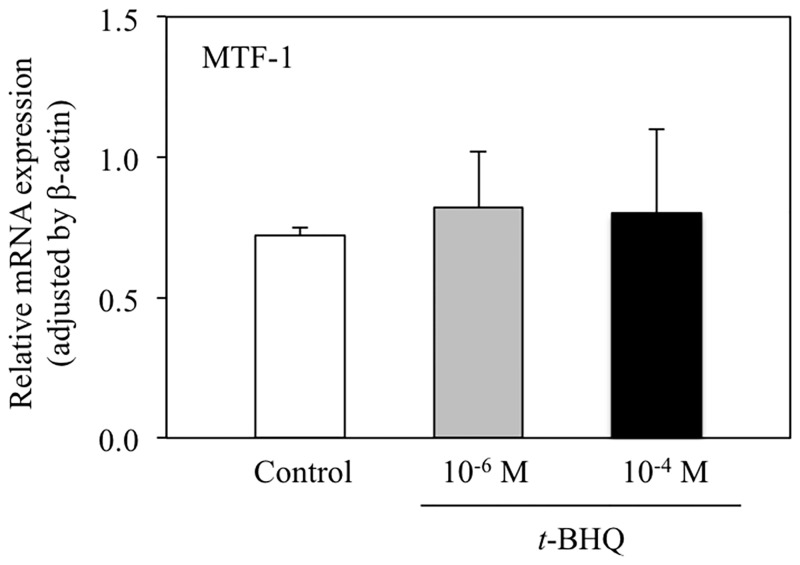
The effect of *t*-BHQ treatment on metal transcriptional factor-1 (MTF-1) mRNA expression. HepG2 cells were treated with the denoted concentration of *t*-BHQ or medium including 0.1% (v/v) DMSO as a control for 6 h. The mRNA level was determined using qRT-PCR with SYBR^®^ Green. Data are normalized to ß-actin expression. Bars represent the mean ± SD of 4 samples.

One of roles of zinc transporters is to maintain intracellular zinc homeostasis, and it is known that their expression is stimulated by alterations in intracellular free zinc levels. The MTF-1-MRE signal transduction pathway plays a critical role in this transcriptional regulation, and it has been shown that ZnT-1 transcription is activated by the MTF-1-MRE signal transduction pathway in response to increased levels of intracellular free zinc [20]. In light of this, we proceeded to investigate whether *t*-BHQ-induced changes in zinc transporter mRNA levels were a consequence of alterations in intracellular zinc levels using an MRE-transcriptional reporter. As shown in Fig 5, *t*-BHQ treatment did not induce a significant change in MRE-luciferase transcription, although the positive control of 0.1 mM ZnSO_4_ did. In accordance with this, the level of MTF-1 mRNA was also unaffected by *t*-BHQ treatment (Fig 6). These results indicate that the MTF-1-MRE signal transduction pathway is not responsible for the altered levels of zinc transporter mRNA observed in *t*-BHQ treated cells.

## Discussion

In this study, we investigated the effect of Nrf2-ARE signaling on zinc transporter mRNA expression. We have shown that ZnT-1, ZnT-3, and ZnT-6 mRNA levels are significantly increased by 10^−4^ M *t*-BHQ treatment, which activates Nrf2-ARE signal transduction (Fig 1). In contrast, ZnT-10 and ZIP-3 mRNA levels were significantly decreased with the same *t*-BHQ treatment. These alterations in mRNA expression were not detected at lower *t*-BHQ concentrations (10^−6^ M; Fig 2) that do not activate Nrf2-ARE signaling. Moreover, the MTF-1-MRE signal transduction pathway, which is activated in response to increased intracellular free zinc levels, was not affected by *t*-BHQ treatment (Fig 5). From these results, we suggest that the Nrf2-ARE signal transduction pathway directly regulates the transcription of some zinc transporters.

It is widely accepted that the Nrf2-ARE signal transduction pathway contributes to the transcriptional regulation of stress response genes. This pathway is activated by cellular stresses, such as oxidative stress [21], and AREs are present in the promoter of numerous enzymes that defend against oxidative and chemical stresses. For example, the NAD(P)H: quinone oxidoreductase [22], glutathione *S*-transferase Ya subunit [23], glutamate-cysteine ligase subunit [24–26], and heme oxygenase-1 genes all contain functional AREs in their promoters. Considering this, it is expected that ZnT-1, ZnT-3 and ZnT-6 are all part of the cellular stress defense system.

Although, the biological function of zinc transporters has not been fully elucidated, ZnT-1 expression has been reported to increase in response to increase levels of intracellular zinc [27]. This suggests that the upregulation of ZnT-1 mRNA forms part of the cellular defense system against heavy metal stress, and lends credence to our observation that ZnT-1 mRNA levels are increased by the Nrf2-ARE signal transduction pathway. However, it is difficult to explain the biological function of the observed *t*-BHQ-induced increase in ZnT-3 and ZnT-6 mRNA expression.

ZnT-6 is reported to be distributed in widespread tissues, where it is localized to the Golgi apparatus and in cytoplasmic vesicles. ZnT-6 has been shown to function with ZnT-5 and ZnT-7 to supply zinc to alkaline phosphatase in the early secretary pathway [28]. The key zinc binding site residues of ZnT-6 are not conserved [29], suggesting that ZnT-6 functions as a modulator of zinc transporter complexes and enhances zinc transporter activity. ZnT-3 is expressed on the membrane of secretory vesicles and is responsible for accumulating zinc into the vesicles for exocytosis [30,31]. However, there are very few reports regarding the transcriptional regulation of ZnT-3 and ZnT-6 expression in response to cellular stress. Further studies are required in order to gain a better understanding of the transcriptional mechanisms through which ZnT-3 and ZnT-6 expression are upregulated by the Nrf2-ARE signal transduction pathway. By the way, it is possible that ZnT-1, ZnT-3, and ZnT-6 mRNA levels are increased to reduce intracellular free zinc levels, which might increase in response to *t*-BHQ-derived disruption of zinc-binding proteins. However, we have demonstrated that *t*-BHQ treatment did not significantly affect MRE-luciferase reporter expression (Fig 5) or MTF-1 mRNA levels (Fig 6), suggesting that *t*-BHQ treatment for 6 h did not increase intracellular free zinc levels.

In contrast to ZnT-1, ZnT-3, and ZnT-6, *t*-BHQ treatment decreased ZnT-10 and ZIP-3 mRNA levels (Fig 1). It has been reported that ZnT-10 is highly expressed in the liver, brain and small intestine [32], and in accordance with other ZnTs, ZnT-10 functions as an efflux transporter to decrease cytoplasmic zinc levels. It has also been suggested that ZnT-10 functions as a manganese transporter because hypermanganesemia has been shown to result from homozygous mutation of the ZnT-10 gene [33,34]. ZIP-3 has been shown to be localized to the plasma membrane and lysosomes, and, in contrast to ZnT-10, transports zinc across the cellular membrane into the cytoplasm, i.e. ZIP-3 influxes zinc from the extracellular space and intracellular organelles [35]. The relationship between Nrf2-ARE signaling activation and ZnT-10 and ZIP-3 mRNA downregulation is not clear. However, a recent report has shown that ZnT-10 mRNA expression is significantly decreased in the frontal cortex of patients with Alzheimer’s disease [9]. In addition, ZIP-3 expression has been shown to be decreased in prostate adenocarcinomas [36]. As such, the downregulation of ZnT-10 and ZIP-3 mRNAs in response to *t*-BHQ may be related to cellular damage caused by *t*-BHQ-induced stress.

## Conclusion

In this study, we have demonstrated that the Nrf2-ARE signal transduction pathway increases ZnT-1, ZnT-3, and ZnT-6 mRNA levels, and decreases ZnT-10 and ZIP-3 mRNA levels. We believe that these alterations in mRNA levels occur at the transcriptional level, however, it is also possible that the non-transcriptional mechanisms, such as mRNA stabilization or degradation, contribute to these changes. Further studies are required to determine whether these alterations in mRNA levels are accompanied by altered zinc transporter protein expression levels. In addition, since it is well-known that the expression pattern of zinc transporters differs in tissue types and cell lines, our results may be a limited phenomena observed in HepG2 cells. However, the transcription mechanisms via Nrf2-ARE signal transduction pathway are nearly identical regardless of tissue types and cell lines. Thus, our results provide new insights into the regulation of zinc transporters, and could aid in our understanding of their biological function and role in disease pathogenesis.

## Supporting Information

S1 Table The accession numbers used for quantitative real-time polymerase chain reaction primer design. The represented symbols are as follows: ZnT, zinc transporter; ZIP, Zrt-, Irt-like proteins; BACT, ß-actin; MTF-1, **metal transcriptional factor-1**; HMOX-1, **heme oxygenase-1.**
(DOCX)
