# Supplementary material for: Nrf2-ARE-Dependent Alterations in Zinc Transporter mRNA Expression in HepG2 Cells
Source: PLoS One. 2016 Nov 3;11(11):e0166100. doi: 10.1371/journal.pone.0166100 (PMC5094758; doi:10.1371/journal.pone.0166100)
Supplement: S1 Table — The represented symbols are as follows: ZnT, zinc transporter; ZIP, Zrt-, Irt-like proteins; BACT, ß-actin; MTF-1, metal transcriptional factor-1; HMOX-1, heme oxygenase-1. (DOCX) [file pone.0166100.s001.docx]

Supporting Information

S1 Table

| Symbol | Accession No. |
| --- | --- |
| ZnT-1 | NM_021194 |
| ZnT-2 | NM_001004434 |
| ZnT-3 | NM_003459 |
| ZnT-4 | NM_013309 |
| ZnT-5 | NM_022902 |
| ZnT-6 | NM_001193513 |
| ZnT-7 | NM_133496 |
| ZnT-8 | NM_173851 |
| ZnT-9 | NM_006345 |
| ZnT-10 | NM_018713 |
| ZIP-1 | NM_014437 |
| ZIP-2 | NM_014579 |
| ZIP-3 | NM_144564 |
| ZIP-4 | NM_017767 |
| ZIP-5 | NM_173596 |
| ZIP-6 | NM_012319 |
| ZIP-7 | NM_006979 |
| ZIP-8 | NM_022154 |
| ZIP-9 | NM_018375 |
| ZIP-10 | NM_001127257 |
| ZIP-11 | NM_001159770 |
| ZIP-12 | NM_001145195 |
| ZIP-13 | NM_001128225 |
| ZIP-14 | NM_001128431 |
| BACT | NM_10277 |
| MTF-1 | NM_005955 |
| HMOX-1 | NM_002133 |
